# Tauroursodeoxycholic acid attenuates neuronal apoptosis via the TGR5/ SIRT3 pathway after subarachnoid hemorrhage in rats

**DOI:** 10.1186/s40659-020-00323-1

**Published:** 2020-12-01

**Authors:** Huihui Wu, Nini Yu, Xia Wang, Yina Yang, Hui Liang

**Affiliations:** 1 Department of Neurology, First Affiliated Hospital, School of Medicine, Zhejiang University, Hangzhou, 310003 China; 2 Department of Neurology, Ninghai People’s Hospital, Ninghai, 315600 China

**Keywords:** TUDCA, Apoptosis, Neuroprotection, Subarachnoid hemorrhage

## Abstract

**Background:**

Neuronal apoptosis plays a critical event in the pathogenesis of early brain injury after subarachnoid hemorrhage (SAH). This study investigated the roles of Tauroursodeoxycholic acid (TUDCA) in attenuate neuronal apoptosis and underlying mechanisms after SAH.

**Methods:**

Sprague–Dawley rats were subjected to model of SAH and TUDCA was administered via the internal carotid injection. Small interfering RNA (siRNA) for TGR5 were administered through intracerebroventricular injection 48 h before SAH. Neurological scores, brain water content, Western blot, TUNEL staining and immunofluorescence staining were evaluated.

**Results:**

TUDCA alleviated brain water content and improved neurological scores at 24 h and 72 h after SAH. TUDCA administration prevented the reduction of SIRT3 and BCL-2 expressions, as well as the increase of BAX and cleaved caspase-3.Endogenous TGR5 expression were upregulated after SAH and treatment with TGR5 siRNA exacerbated neurological outcomes after SAH and the protective effects of TUDCA at 24 h after SAH were also abolished by TGR5 siRNA.

**Conclusions:**

Our findings demonstrate that TUDCA could attenuated neuronal apoptosis and improve neurological functions through TGR5/ SIRT3 signaling pathway after SAH. TUDCA may be an attractive candidate for anti-apoptosis treatment in SAH.

## Background

Aneurysmal subarachnoid hemorrhage (SAH) is a devastating disease with high mortality and morbidity [1]. Studies suggest that the pathophysiological events that during the first 72 h following SAH, are the major factor which is closely related to prognosis [2]. There is compelling evidence to suggest that neuronal apoptosis play a pivotal role in the development of early brain injury after SAH and apoptosis inhibition by pharmacological treatment could protect against brain injury after SAH [3].

Tauroursodeoxycholic acid (TUDCA) is an endogenous hydrophilic bile acid used clinically to treat certain liver diseases [4]. Previous studies have showed that TUDCA can significantly reduce brain injury and improve neurological function associated with acute hemorrhagic stroke or acute ischemic stroke in rats through inhibiting neuronal apoptosis [5, 6]. TGR5 is a plasma membrane-bound G protein-coupled bile acid receptor, which has varied levels of expression in different tissues including brain [7, 8].

TUDCA may activate TGR5-mediated signaling to reduce DNA damage and improve embryo development after ultraviolet light exposure [9]. The effects of TUDCA on insulin secretion were mimicked by the specific TGR5 agonist INT-777 [10]. In central nervous system research, studies identified that TGR5 activation alleviated brain injuries in experimental autoimmune encephalomyelitis, hepatic encephalopathy and Aβ1-42-induced neurotoxicity [11–13]. Yanguas-Casás etal found that the anti-inflammatory effect of TUDCA on microglia are mediated by TGR5 [14]. However, the anti-apoptotic property of TUDCA by interacting with TGR receptor has not been reported in the setting of SAH.Sirtuin3 (SIRT3), a member of the silent information regulator 2 (Sir2) family of proteins located in mitochondria, confers protection against neuronal ischemia by inhibiting apoptosis [15]. Recent research indicated that activating TGR5 induced increased activity of SIRT3 in diabetes- and obesity-related kidney disease [16].

In the current study, we hypothesized that (1) TUDCA provides neuroprotective effects after SAH; (2) TUDCA attenuates apoptosis by activating TGR5 signaling after SAH.

## Results

### General observations and mortality rate

A total of 254 rats were used and 224 rats underwent SAH induction. There were no deaths in sham group. For groups of SAH, the mortality rate was 11.6% (26 of 224) (Additional file 1: Table S1). According to the SAH grading score, 7 rats with mild SAH were excluded from this experiment.

### TUDCA improved neurobehavioral functions and reduced brain edema at 24 h and 72 h after SAH

**Fig. 1:**
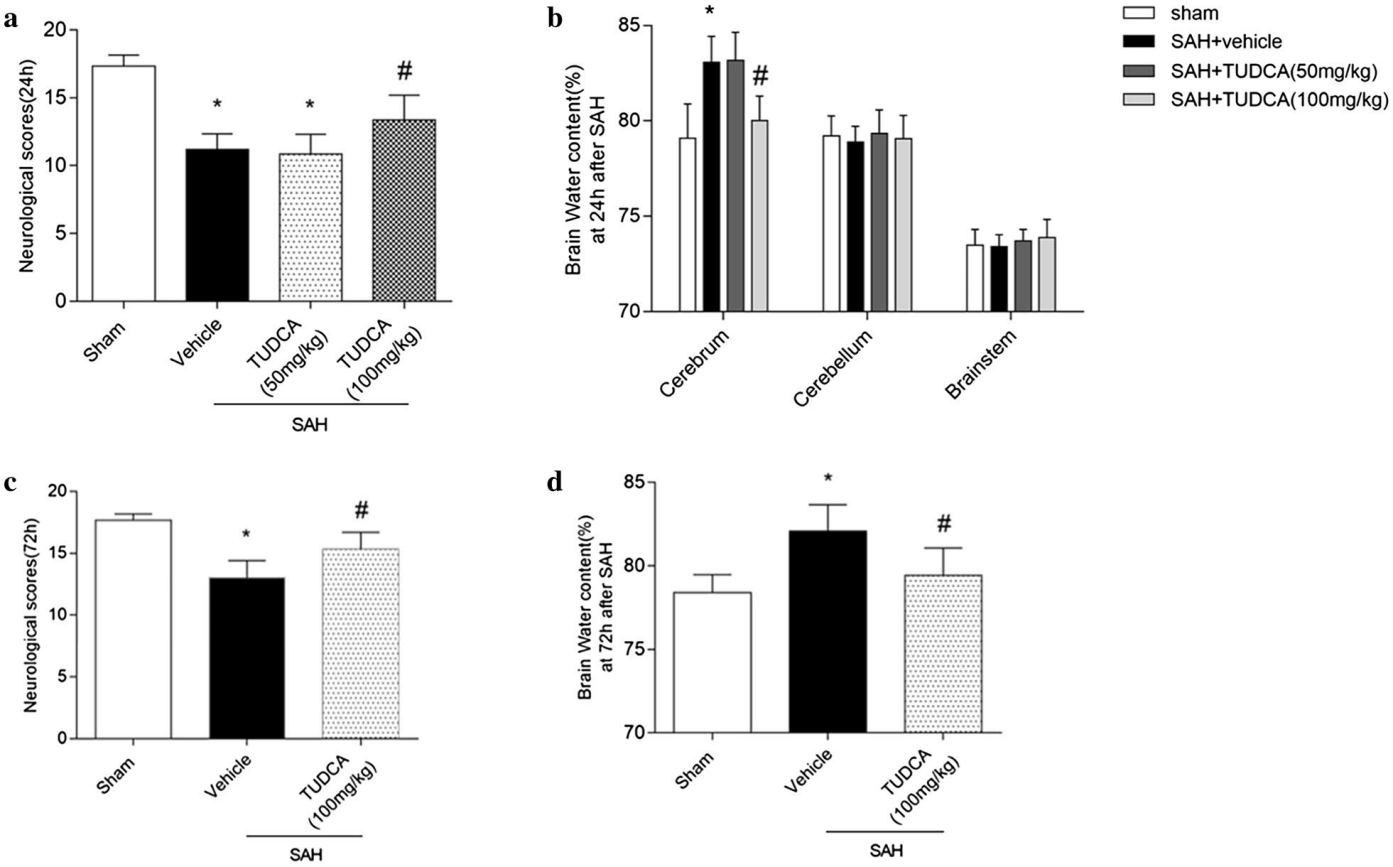
The protective role of TUDCA after subarachnoid hemorrhage (SAH). **a**, **c** TUDCA attenuated neurological deficits at 24 h or 72 h following SAH. **b**, **d** TUDCA reduced brain edema at 24 h or 72 h following SAH. Data are expressed as the mean ± SD (n = 6 for each group). *P < 0.05, vs. sham group, ^#^*P* < 0.05, vs. SAH + vehicle group

The neurological scores of modified Garcia were significantly reduced at 24 h in the SAH + vehicle group (*P* < 0.05 versus Sham) (Fig. 1a). The 100 mg/kg TUDCA-treated rats presented with improved neurobehavioral score (*P* < 0.05versus Vehicle), (Fig. 1a), but not with the 50 mg/kg TUDCA-treated rats. SAH significantly increased the brain edema in the SAH + vehicle group at 24 h when compared to the sham group(*P* < 0.05) (Fig. 1b). Treatment with high doses of TUDCA reduced brain edema (*P* < 0.05 versus SAH + vehicle) (Fig. 1b). Administration of TUDCA(100 mg/kg) also improved neurological function and decreased brain edema at 72 h after injury (*P* < 0.05 versus SAH + vehicle) (Fig. 1 c, d).As illustrated in Additional file 1: Figure S1a, neurological recovery was also observed when TUDCA was administered at 6 h or 12 h after SAH onset, but not when the administration was delayed to 24 h. Based on the outcome study, the dose of 100 mg/kg TUDCA administration at 1 h was used for the rest of the experiments.

### Endogenous TGR5 receptor were increased at 24 hours after SAH

**Fig. 2:**
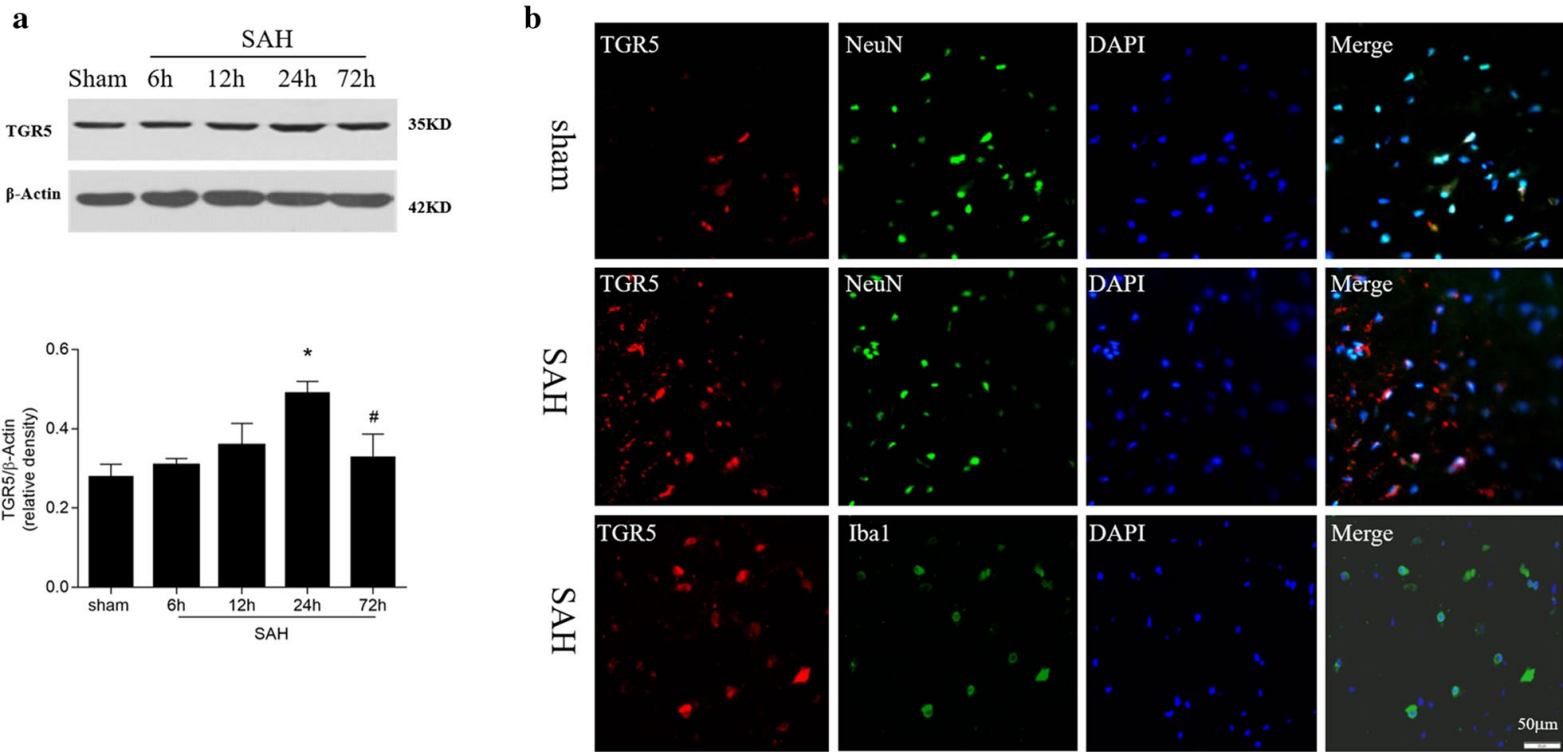
Expressions of TGR5 after experimental subarachnoid hemorrhage (SAH). **a **Representative western blot images and quantitative analyses of TGR5 time course from left hemisphere after SAH. n = 6 per group, *P < 0.05 vs sham group, ^#^*P* < 0.05, vs. SAH 24 h. **b** Double immunofluorescence staining for TGR5 (red) in neurons (NeuN, green),microglia (Iba-1, green) following SAH. n = 6 per group. Bars represent mean ± SEM. Scale bar, 50 μm. Iba-1 indicates ionized calcium binding adaptor molecule 1; *NeuN* neuronal nuclear

As shown in Fig. 2a, TGR5 expression began to increase at 12 h and reached peak at 24 h but declined at 72 h after SAH group (*P* < 0.05 versus Sham).Double immunofluorescence staining showed that TGR5 was upregulated in neurons in the ipsilateral cortex at 24 h after SAH and was also localized in microglia (Fig. 2b).

### TUDCA inhibited the cortical neuronal apoptosis

**Fig. 3:**
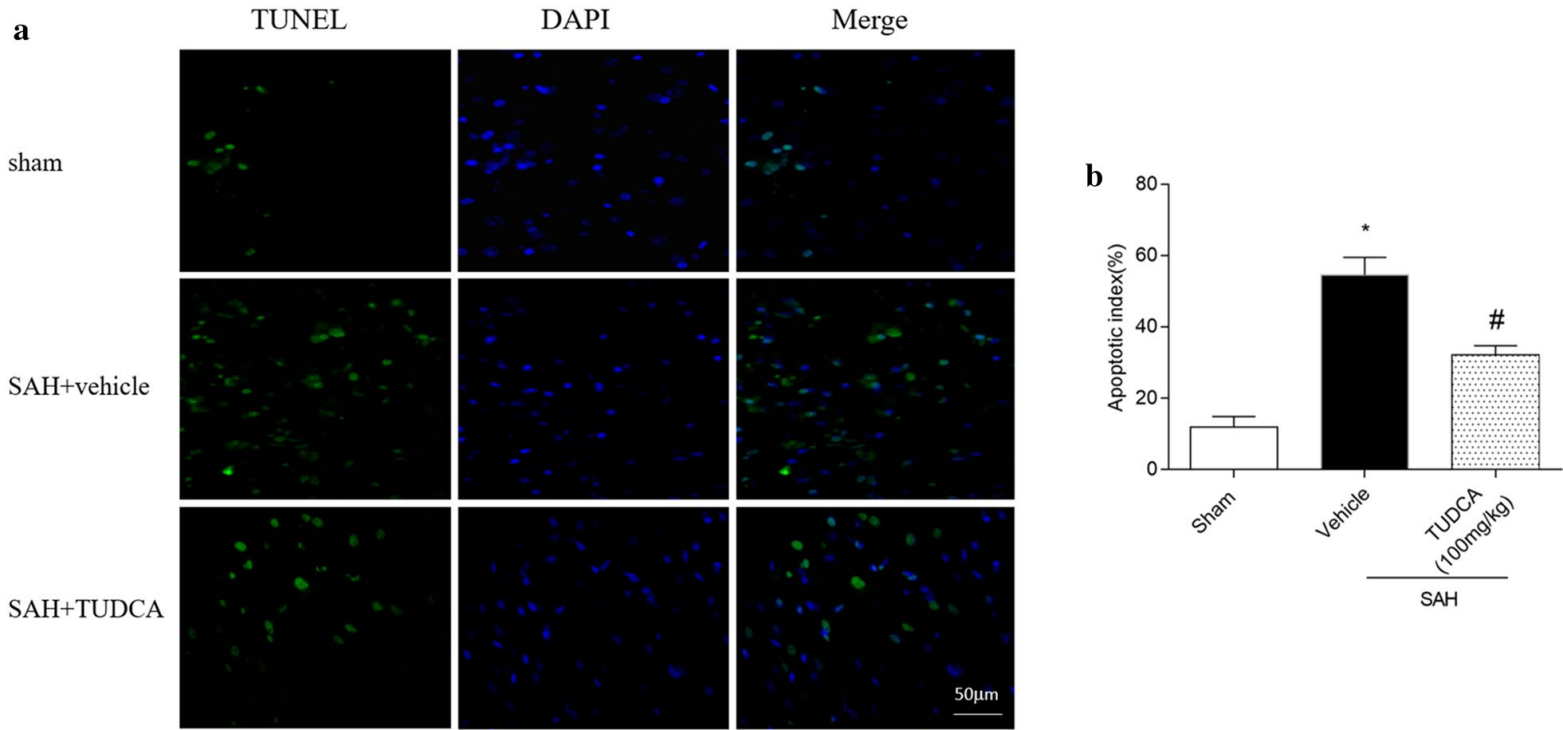
TUDCA reduced apoptosis following subarachnoid hemorrhage (SAH). **a** Representative microphotographs of TUNEL-positive neurons in the ipsilateral cortex. **b** Quantified number of apoptotic cells. Data are expressed as the mean ± SD (n = 6 for each group). *P < 0.05, vs. sham group, ^#^*P* < 0.05, vs. SAH + vehicle group. Bars represent mean ± SEM. Scale bar, 50 μm

Compared with sham group, the numbers of TUNEL-positive cells were significantly higher in the SAH + vehicle group while TUDCA distinctly decreased the number of positive cells (Fig. 3a, b).

**Fig. 4:**
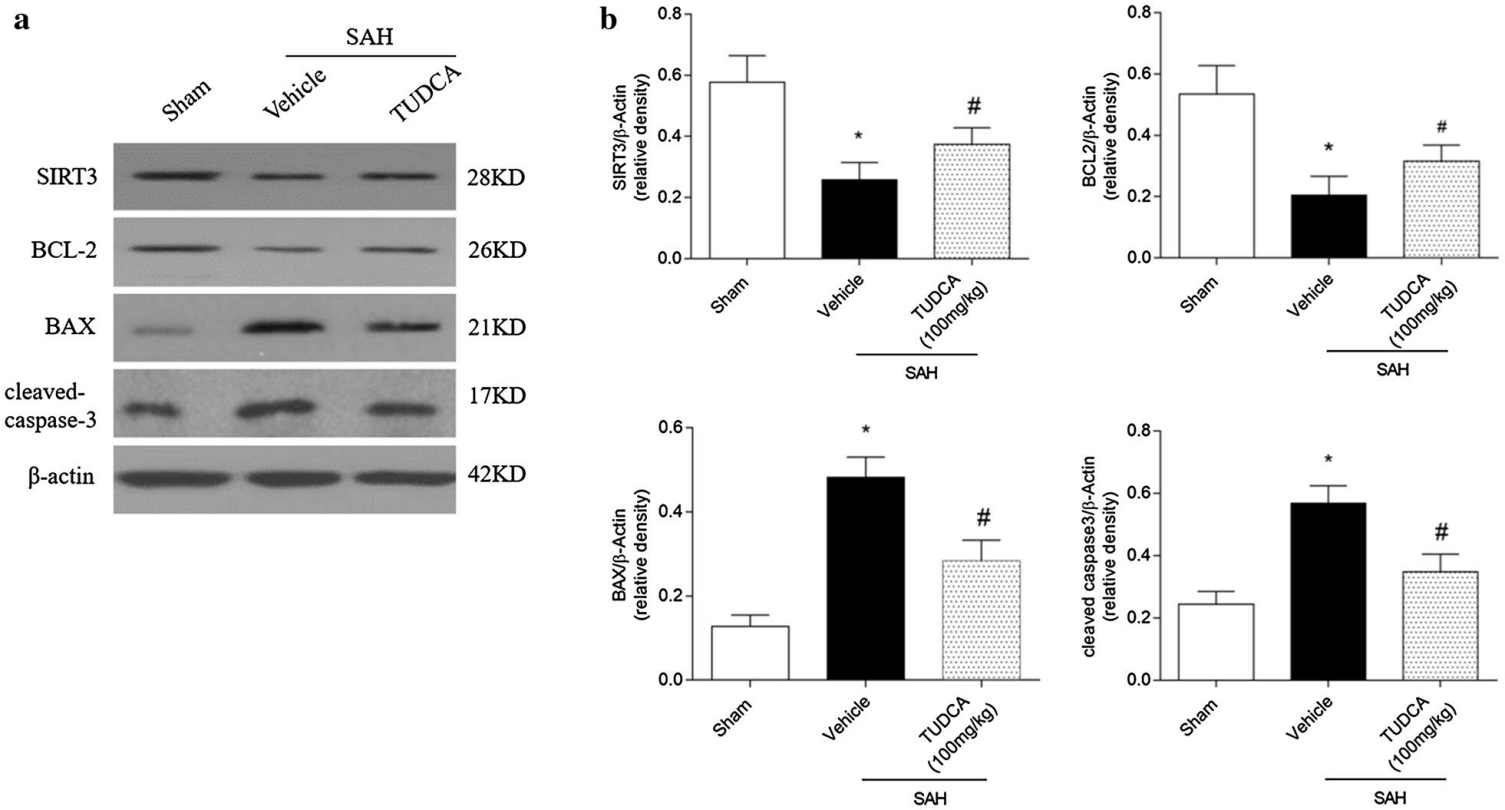
The effects of TUDCA on the protein expression of SIRT3, BCL-2, BAX and cleaved caspase-3 after subarachnoid hemorrhage (SAH). **a** Representative Western blot images. **b** The relative density of protein expressions. n = 6 per group. *P < 0.05 vs sham, ^#^*P* < 0.05 vs SAH + vehicle. Bars represent mean ± SEM. Scr siRNA, scramble siRNA

Western blot showed that SAH reduced the expressions of SIRT3 and BCL-2 when compared to sham group (P < 0.05) and TUDCA prevented the reductions (P < 0.05) (Fig. 4 a, b). Furthermore, BAX and cleaved caspase3 were increased following SAH (P < 0.05) and TUDCA reversed these effects (P < 0.05) (Fig. 4 a, b).

### TRG5siRNA reversed the neuroprotection of TUDCA

**Fig. 5:**
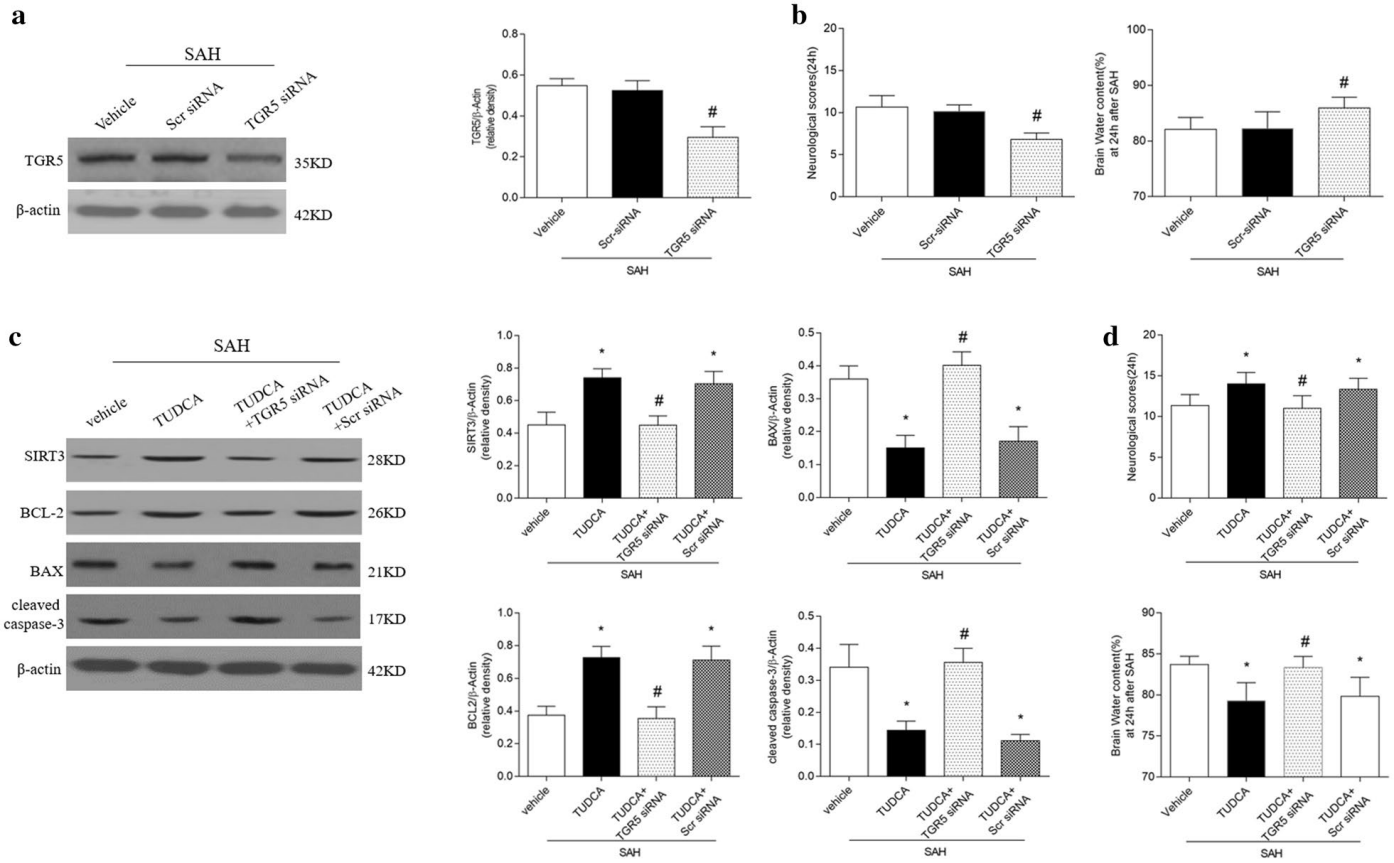
The knockdown efficiency of TGR5 siRNA. Representative Western blots (**a**), and TGR5 knockout worsened neurobehavioral deficits, exacerbated brain water content (**b**). Knockdown TGR5 abolished the anti-apoptotic effect of TUDCA after subarachnoid hemorrhage (SAH). **c** Representative Western blots and quantitative analyses of SIRT3, BCL-2, BAX and cleaved caspase-3. Quantified neurological scores and brain water content (**d**), n = 6 per group. *P < 0.05 vs sham, ^#^*P* < 0.05 vs SAH + vehicle. Scr siRNA, scramble siRNA

To further assess the role of TUDCA in SAH, TGR5 siRNA was administered by ICV injection to knockdown endogenous TGR5. Western blot showed that TGR5 expression was partially prevented by TGR5 siRNA (Fig. 5a). Silencing of endogenous TGR5 significantly aggravated neurological impairments (Fig. 5b).

TGR5 siRNA significantly reversed the effect of TUDCA on expression of SIRT3, BCL-2、Bax and cleaved caspase-3 when compared with scramble siRNA at 24 h after SAH (*P* < 0.05) (Fig. 5c). Administration of TGR5 siRNA significantly abolished the protective effect of TUDCA on neurological deficits and brain edema at 24 h after SAH (*P* < 0.05 versus SAH + TUDCA) (Fig. 5d).

## Discussion

In the present study, we described the possible mechanism of anti-apoptosis of TUDCA after SAH in rats. Our data demonstrated that TGR5 were upregulated after SAH. TUDCA reduced neuronal apoptosis, decreased brain edema and thereby alleviated stroke outcome after SAH. TUDCA prevented the reduction of SIRT3 and BCL-2 expressions and reversed the increasing of BAX and cleaved caspase-3. Furthermore, knockdown TGR5 by siRNA abolished the beneficial effects of TUDCA, which were associated with increased expressions of BAX and cleaved caspase-3. Taken together, our study suggested that TUDCA attenuated apoptosis and brain injury after SAH, which is at least in part mediated by TGR5/ SIRT3 signaling cascade pathway.

TUDCA is an endogenous hydrophilic bile acid used clinically to treat certain liver diseases [24]. It is formed by the conjugation of ursodeoxycholic acid (UDCA) with taurine. TUDCA is neuroprotective not only in pharmacologic and transgenic animal models of Huntington disease [25], but also for acute ischemic stroke and intracerebral hemorrhage [6]. In our research, we found that high dosage of TUDCA treatment attenuated the neurological deficit and the SAH-induced brain edema. Taken together, these founding suggest that TUDCA could provide neuroprotection following SAH.

TUDCA has been demonstrated to exert its cytoprotective activity by preventing apoptosis. TUDCA may prevent palmitate-induced apoptosis in rat pancreatic β-cell line INS-1 and to evoke beneficial effects on acinar cells in the experimental model of acute pancreatitis mostly [26]. Beneficial effects of TUDCA have also been suggested in studies of diabetic retinopathy through against high glucose-dependent apoptosis [27]. In neurological disorders, TUDCA not only has been shown to inhibit apoptosis induced by several stimuli in neuronal cells in vitro, but also to play a cytoprotective role in animal models, such as Alzheimer’s disease (AD), Parkinson’s disease, Huntington’s disease (HD) and acute hemorrhagic stroke [28]. Our observations showed that administration of TUDCA significantly prevented the reduction of antiapoptotic markers, and reversed the increasing of apoptotic markers and the number for TUNNEL-positive neurons.

The exact mechanisms of TUDCA- mediated neuroprotection in SAH have been remained unclear. Results indicated that TUDCA is neuroprotective in neurological diseases through anti-neuroinflammation, which is involved in TGR5-mediated signaling [14]. TGR5, as a bile acid receptor, has been shown roles in alleviating the liver ischemia/reperfusion-related inflammation and protecting hepatocytes from ischemia/reperfusion-related apoptosis [29]. Overexpression of TGR5 significantly improved cardiomyocyte cell proliferation, alleviated apoptosis rate [30]. INT-777, a specific TGR5 agonist, alleviates Aβ1–42-induced neuronal apoptosis in the hippocampus and frontal cortex [13]. SIRT3, a NAD-dependent protein deacetylase, belongs to the silent information regulator 2 family. Several evidences have confirmed that TGR5 is a key regulator of SIRT3 in human kidney samples [16]. SIRT3 might play an important neuroprotective role during early brain injury following SAH [31]. SIRT3 apoptotic signaling pathways play important role in puerarin induced neuroprotection in SAH [32]. In our research, we observed that endogenous SIRT3 expression was decreased at 24 h after SAH.TUDCA prevented the expressions of SIRT3 while TGR5 siRNA reversed the effect of TUDCA. Furthermore, our data demonstrated that TGR5 knockdown significantly reverses the neuro-protection of TUDCA on stroke outcomes, as well as increasing BCL-2 and decreasing BAX and cleaved caspase-3 expression. This finding supports the notion that the TGR5/SIRT3 signaling pathway plays a role in anti-neuronal apoptotic function of TUDCA after SAH.

There are some limitations in the present research. First, TUDCA may alleviated inflammation and we only focused on the neuroprotective effects on anti-apoptosis after SAH, but further studies are needed to explore other effects of TDUCA and its possible mechanisms. Second, our study cannot rule out the possibility that blood–brain barrier preserving may be involved in the neuroprotective role of TDUCA in brain injury after SAH.

## Conclusions

In conclusion, our findings indicated that TUDCA could attenuate neuronal apoptosis and improve neurological outcome after SAH, which was probably mediated by activation of TGR5/SIRT3 signaling cascade and subsequent suppression of apoptotic proteins expressions. Thus, TUDCA may be an attractive candidate for anti-apoptosis treatment in SAH.

## Methods

### Animal preparation

All the protocols used in this study were approved by the Institutional Animal Care and Use Committee at the First Affiliated Hospital, School of Medicine, Zhejiang University. Adult male Sprague–Dawley rats weighing 280–320 g were randomly assigned to the following 10 groups: sham, SAH, SAH + vehicle, SAH + TUDCA (50 mg/kg), SAH + TUDCA (100 mg/kg), SAH + scramble siRNA, SAH + TGR5 siRNA, SAH + scramble siRNA + TUDCA (100 mg/kg), SAH + TGR5 siRNA + TUDCA (100 mg/kg) and sham + TUDCA (100 mg/kg). The rats were raised on a 12 h dark–light cycle circumstance with free access to food and water.

### Preparing rat SAH model

The endovascular perforation was performed as previously described with modifcations [17]. Anesthesia was induced with sodium pentobarbital (80 mg/kg). After anesthesia of intraperitoneal injection in mice, the left common carotid artery (CCA), internal carotid artery (ICA) and external carotid artery (ECA) were surgically exposed. A sharpened 3.0 monofilament nylon suture was inserted rostrally into the left ICA from ECA stump until resistance was felt and then pushed 3 mm further penetrating the ICA near the bifurcation with the middle cerebral artery (MCA). The suture was then withdrawn to induce SAH. The sham-operated group underwent the same procedure without an endovascular puncture. After removal of the suture, the skin incision was sutured, and the rats were kept at approximately 37 °C on an electric heating blanket until completely recovered from anesthetic.

### SAH grading.

SAH grade was determined by the high-resolution images of the blood clots in the basal cisterns, as previously described [18]. According to the total score, it can be rated as the following three groups: 0–7 points: mild SAH, 8–12 points: moderate SAH, 13 to 18 points: severe SAH. Rats with the grade < 8 at 24 h after SAH were excluded from this study.

### Brain water content

Brain water content was evaluated at 24 and 72 h after SAH. The brains were separated into cerebrum, cerebellum, and brainstem. Each part was weighed immediately after removal (wet weight) and then dried in an oven at 80 °C for 72 h (dry weight). After that, the percentage of brain water content was calculated as [(wet weight—dry weight)/wet weight] × 100% [19].

### TUDCA administration

Saline or TUDCA (Baoji bioscience,China), dissolved in phosphate buffer, pH 7.4 at 400 mg/ml, was slowly injected into the internal carotid at 1 ml/kg of bodyweight (bw). TUDCA at 50 or 100 mg/kg of bw was administered 1 h after SAH model.

### TUNEL staining

TUNEL assay was conducted as previously described with modifications [20]. TUNEL reaction mixture (50 μL) was added on each sample, and the slides were incubated in a humidified dark chamber for 60 min at 37 °C. The slides were then incubated with DAPI for 5 min at room temperature in the dark to stain the nuclei, followed by imaging with a fluorescence microscope. Apoptotic index was expressed as the ratio of the number of TUNEL‐positive neurons to the total number of neurons in the field of view.

### Intracerebroventricular siRNA injection

Intracerebroventricular (i.c.v) drug administration was performed was performed as described previously [21]. TGR5-siRNA, or scramble-siRNA (OriGene Technologies) were dissolved in RNase-free H2O firstly. Then, TGR5 TGR5-siRNA, or scramble-siRNA was diluted with in vivo transfection reagent and mixed gently. Finally, the mixture (100 pmol in 2 μL) was inserted into the right lateral ventricle using a Hamilton microsyringe under the guidance of a stereotaxy instrument. The stereotaxic coordinates were 1.5 mm posterior, 0.9 mm lateral, and 3.3 mm below the horizontal plane of the bregma. The needle was retracted 5 min after the injection and the burr hole was sealed with bone wax. The SAH model was established 48 h later.

### Immunofluorescence staining

Double fluorescence staining was performed as described previously [22]. The rats were deeply anesthetized at 24 h post-SAH and were transcardially perfused with 60-mlice-cold PBS followed by 60 ml of 10% paraformaldehyde. The whole brains were collected and then fixed in 10% paraformaldehyde for 24 h followed by 30% sucrose solution for another 72 h. Frozen coronal slices (10 μm) were sectioned and permeabilized with 0.3% Triton X-100 in PBS for 30 min. Sections were blocked with 5% donkey serum for 1 h and incubated at 4 °C overnight with primary antibodies: rabbit anti-TGR5 (1:200, Abcam), goat polyclonal anti-Iba-1 (1:200 Abcam) and anti-NeuN (1:200 Abcam), followed by incubation with appropriate fluorescence-conjugated secondary antibody for 2 h at room temperature. The slides were then read using a confocal microscope (Olympus, Tokyo, Japan).

### Western blot analysis

Western blot tests were performed as previously described [23]. Brains were removed, and tissues from the ipsilateral/left cerebral cortex were dissected rapidly and were frozen in liquid nitrogen at -80℃. After sample preparation, equal amounts of protein samples (50 μg) were loaded onto each lane of SDS-PAGE gel. After electrophoresis, the samples were transferred onto a nitrocellulose membrane, which was blocked with a blocking solution for 2 h. The membrane was incubated at 4 °C overnight with the following primary antibody: rabbit anti-TGR5 (1:1000, Abcam), rabbit anti-SIRT3 (1:1000, Abcam), rabbit anti-BCL-2 antibody (1:500; Santa Cruz), rabbit anti-BAX antibody (1:500; Santa Cruz), rabbit anti-cleaved caspase-3 antibody (1:100; Santa Cruz). β-actin was used as an internal loading control. The secondary antibodies were all from Santa Cruz Biotechnology. Blot bands were quantified by densitometry using ImageJ software (ImageJ 1.4; NIH, Bethesda, MD).

### Statistical analysis

Data were analyzed using SigmaPlot 11.0 and GraphPad Prism 6 (GraphPad software, San Diego, CA), expressed as means ± standard deviation (SD). Data from different groups were compared using 1-way ANOVA followed by post hoc Tukey tests. Non-parametric data (neurological scores) were analyzed with the Kruskal–Wallis test followed by Dunn’s post-hoc. Significant differences were accepted when a value of p < 0.05.

## Electronic supplementary material

Below is the link to the electronic supplementary material.

**Additional file 1: Table S1.** Animal number (Survival/total) in each group.** Figure S1.** (a) The effect of TUDCA in different administration time on neurological damage. n = 6 per group. *P<0.001 vs SAH+vehicle. (b–d) Representative Western blot images and quantitative analyses of SIRT3 and BAX in sham group and sham+TUDCA group. n = 6 per group.

## Data Availability

All data generated or analyzed during this study are included in this published article.

## Abbreviations

SAH: Subarachnoid hemorrhage
TUDCA: Tauroursodeoxycholic acid
SIRT3: Sirtuin3

## References

[1] Connolly ES, Rabinstein AA, Carhuapoma JR (2012). Guidelines for the management of aneurysmal subarachnoid hemorrhage: a guideline for healthcare professionals from the American Heart Association/American Stroke Association. Stroke.

[2] Hasegawa Y, Suzuki H, Sozen T (2011). Apoptotic mechanisms for neuronal cells in early brain injury after subarachnoid hemorrhage. Acta Neurochir Suppl.

[3] Serrone JC, Maekawa H, Tjahjadi M (2015). Aneurysmal subarachnoid hemorrhage: pathobiology, current treatment and future directions. Expert Rev Neurother.

[4] Amaral JD, Viana RJ, Ramalho RM (2009). Bile acids: regulation of apoptosis by ursodeoxycholic acid. J Lipid Res.

[5] Rodrigues CM, Sola S, Nan Z (2003). Tauroursodeoxycholic acid reduces apoptosis and protects against neurological injury after acute hemorrhagic stroke in rats. Proc Natl Acad Sci USA.

[6] Rodrigues CM, Spellman SR, Solá S (2002). Neuroprotection by a bile acid in an acute stroke model in the rat. J Cereb Blood Flow Metab.

[7] Kawamata Y, Fujii R, Hosoya M (2003). A G protein-coupled receptor responsive to bile acids. J Biol Chem..

[8] Keitel V, Görg B, Bidmon HJ (2010). The bile acid receptor TGR5 (Gpbar-1) acts as a neurosteroid receptor in brain. Glia.

[9] Dicks N, Gutierrez K, Currin L (2020). Tauroursodeoxycholic acid acts via TGR5 receptor to facilitate DNA damage repair and improve early porcine embryo development. Mol Reprod Dev.

[10] Vettorazzi JF, Ribeiro RA, Borck PC (2016). The bile acid TUDCA increases glucose-induced insulin secretion via the cAMP/PKA pathway in pancreatic beta cells. Metabolism.

[11] Lewis ND, Patnaude LA, Pelletier J (2014). A GPBAR1 (TGR5) small molecule agonist shows specific inhibitory effects on myeloid cell activation in vitro and reduces experimental autoimmune encephalitis (EAE) in vivo. PLoS ONE.

[12] McMillin M, Frampton G, Tobin R (2015). TGR5 signaling reduces neuroinflammation during hepatic encephalopathy. J Neurochem.

[13] Wu X, Lv YG, Du YF (2018). Neuroprotective effects of INT-777 against Aβ1-42-induced cognitive impairment, neuroinflammation, apoptosis, and synaptic dysfunction in mice. Brain Behav Immun.

[14] Yanguas-Casás N, Barreda-Manso MA, Nieto-Sampedro M (2017). TUDCA: an agonist of the bile acid receptor GPBAR1/TGR5 with anti-inflammatory effects in microglial cells. J Cell Physiol.

[15] Feng L, Gao J, Liu Y (2018). Icariside II alleviates oxygen-glucose deprivation and reoxygenation-induced PC12 cell oxidative injury by activating Nrf2/SIRT3 signaling pathway. Biomed Pharmacother.

[16] Wang XX, Wang D, Luo Y (2018). FXR/TGR5 dual agonist prevents progression of nephropathy in diabetes and obesity. J Am Soc Nephrol.

[17] Sehba FA, Flores R, Muller A (2010). Adenosine A(2A) receptors in early ischemic vascular injury after subarachnoid hemorrhage. Lab Invest J Neurosurg.

[18] Sugawara T, Ayer R, Jadhav V (2008). A new grading system evaluating bleeding scale in filament perforation subarachnoid hemorrhage rat model. J Neurosci Methods.

[19] Zhang X, Wu Q, Lu Y (2018). Cerebroprotection by salvianolic acid B after experimental subarachnoid hemorrhage occurs via Nrf2- and SIRT1-dependent pathways. Free Radic Biol Med..

[20] Chen J, Xuan Y, Chen Y (2019). Netrin-1 alleviates subarachnoid haemorrhage-induced brain injury via the PPARγ/NF-KBsignalling pathway. J Cell Mol Med..

[21] Dang B, Li H, Xu X (2015). Cyclophilin A/cluster of differentiation 147 interactions participate in early brain injury after subarachnoid hemorrhage in rats. Crit Care Med.

[22] Dang B, Li H, Xu X (2015). Cyclophilin A/cluster of differentiation 147 interactions participate in early brain injury aftersubarachnoid hemorrhage in rats. Crit Care Med.

[23] Kristian T, Balan I, Schuh R (2011). Mitochondrial dysfunction and nicotinamide dinucleotide catabolism as mechanisms of cell death and promising targets for neuroprotection. J Neurosci Res..

[24] Kim SH, Kwon D, Lee S (2019). Polyhexamethyleneguanidine Phosphate-Induced Cytotoxicity in Liver Cells Is Alleviated by Tauroursodeoxycholic Acid (TUDCA) via a Reduction in Endoplasmic Reticulum Stress. Cells.

[25] Keene CD, Rodrigues CM, Eich T (2002). Tauroursodeoxycholic acid, a bile acid, is neuroprotective in a transgenic animal model of Huntington's disease. Proc Natl Acad Sci U S A.

[26] Seyhun E, Malo A, Schäfer C (2011). Tauroursodeoxycholic acid reduces endoplasmic reticulum stress, acinar cell damage, and systemic inflammation in acute pancreatitis. Am J Physiol Gastrointest Liver Physiol.

[27] Gaspar JM, Martins A, Cruz R (2013). Tauroursodeoxycholic acid protects retinal neural cells from cell death induced by prolonged exposure to elevated glucose. Neuroscience.

[28] Kusaczuk M (2019). Tauroursodeoxycholate-bile acid with chaperoning activity: molecular and cellular effects and therapeutic perspectives. Cells.

[29] Yang H, Zhou H, Zhuang L (2017). Plasma membrane-bound G protein-coupled bile acid receptor attenuates liver ischemia/reperfusion injury via the inhibition of toll-like receptor 4 signaling in mice. Liver Transpl.

[30] Li J, Cheng R, Wan H (2020). Overexpression of TGR5 alleviates myocardial ischemia/reperfusion injury via AKT/GSK-3β mediated inflammation and mitochondrial pathway. Biosci Rep..

[31] Huang W, Huang Y, Huang RQ (2016). SIRT3 expression decreases with reactive oxygen species generation in rat cortical neurons during early brain injury induced by experimental subarachnoid hemorrhage. Biomed Res Int.

[32] Zhang Y, Yang X, Ge X (2019). Puerarin attenuates neurological deficits via Bcl-2/Bax/cleaved caspase-3 and Sirt3/SOD2 apoptotic pathways in subarachnoid hemorrhage mice. Biomed Pharmacother.

