# Supplementary material for: Tauroursodeoxycholic acid attenuates neuronal apoptosis via the TGR5/ SIRT3 pathway after subarachnoid hemorrhage in rats
Source: Biol Res. 2020 Dec 1;53:56. doi: 10.1186/s40659-020-00323-1 (PMC7709410; doi:10.1186/s40659-020-00323-1)
Supplement: Supplementary file 1 — Additional file 1: Table S1. Animal number (Survival/total) in each group. Figure S1. (a) The effect of TUDCA in different administration time on neurological damage. n = 6 per group. *P<0.001 vs SAH+vehicle. (b–d) Representative Western blot images and quantitative analyses of SIRT3 and BAX in sham group and sham+TUDCA group. n = 6 per group. [file 40659_2020_323_MOESM1_ESM.docx]

Full title: Tauroursodeoxycholic acid attenuates neuronal apoptosis via the TGR5/ SIRT3 pathway after subarachnoid hemorrhage in rats

Authors’ names: Huihui Wu^1^, Nini Yu^1^,Xia Wang^1^, Yina Yang^2^, Hui Liang^1^

**Table S**1. Animal number (Survival/total) in each group.

| **Group** | **Neurological Score and**  **Brain water content** | **IF** | **Tunnel**  **Staining** | **WB** |
| --- | --- | --- | --- | --- |
| Sham | 12/12 | 6/6 |  | 12/12 |
| SAH |  | 6/7 |  | 24/28 |
| SAH+Vehicle | 30/34 |  | 6/7 | 18/20 |
| SAH+TUDCA (50 mg/kg) | 6/7 |  |  |  |
| SAH+TUDCA (100 mg/kg) | 36/40 |  | 6/7 | 12/13 |
| SAH+Scr siRNA | 6/6 |  |  | 6/7 |
| SAH+TGR5 siRNA | 6/7 |  |  | 6/7 |
| SAH+TUDCA+Scr siRNA | 6/7 |  |  | 6/7 |
| SAH+TUDCA+TGR5 siRNA | 6/7 |  |  | 6/7 |
| Sham+TUDCA |  |  |  | 6/6 |
| total | 108/120 | 12/13 | 12/14 | 96/107 |

**IF: Immunofluorescence staining; WB: Western blot;** **7 excluded animals were not added in this table**

**Figure S1** (a) The effect of TUDCA in different administration time on neurological damage. n=6 per group. **P*<0.001 vs SAH+vehicle. (b-d) Representative Western blot images and quantitative analyses of SIRT3 and BAX in sham group and sham+TUDCA group. n=6 per group.

**

**
